# Neighbourhood immigration, health care utilization and outcomes in patients with diabetes living in the Montreal metropolitan area (Canada): a population health perspective

**DOI:** 10.1186/s12913-015-0824-1

**Published:** 2015-04-09

**Authors:** Alain Vanasse, Josiane Courteau, Maria Gabriela Orzanco, Patrick Bergeron, Alan A Cohen, Théophile Niyonsenga

**Affiliations:** 1 Département de médecine de famille et de médecine d’urgence, Faculté de médecine et des sciences de la santé, Université de Sherbrooke, 3001, 12th Avenue N, Sherbrooke, QC J1H 5N4 Canada; 2 Groupe de recherche PRIMUS, Centre de recherche CHUS, Sherbrooke, QC Canada; 3 Spatial Epidemiology and Evaluation Research Group, Sansom Institute for Health Research, University of South Australia, Adelaide, SA Australia

**Keywords:** Immigration, Diabetes, Deprivation indices, Health inequality, Health outcomes, Neighbourhood

## Abstract

**Background:**

Understanding health care utilization by neighbourhood is essential for optimal allocation of resources, but links between neighbourhood immigration and health have rarely been explored. Our objective was to understand how immigrant composition of neighbourhoods relates to health outcomes and health care utilization of individuals living with diabetes.

**Methods:**

This is a secondary analysis of administrative data using a retrospective cohort of 111,556 patients living with diabetes without previous cardiovascular diseases (CVD) and living in the metropolitan region of Montreal (Canada). A score for immigration was calculated at the neighbourhood level using a principal component analysis with six neighbourhood-level variables (% of people with maternal language other than French or English, % of people who do not speak French or English, % of immigrants with different times since immigration (<5 years, 5–10 years, 10–15 years, 15–25 years)). Dependent variables were all-cause death, all-cause hospitalization, CVD event (death or hospitalization), frequent use of emergency departments, frequent use of general practitioner care, frequent use of specialist care, and purchase of at least one antidiabetic drug. For each of these variables, adjusted odds ratios were estimated using a multilevel logistic regression.

**Results:**

Compared to patients with diabetes living in neighbourhoods with low immigration scores, those living in neighbourhoods with high immigration scores were less likely to die, to suffer a CVD event, to frequently visit general practitioners, but more likely to visit emergency departments or a specialist and to use an antidiabetic drug. These differences remained after controlling for patient-level variables such as age, sex, and comorbidities, as well as for neighbourhood attributes like material and social deprivation or living in the urban core.

**Conclusions:**

In this study, patients with diabetes living in neighbourhoods with high immigration scores had different health outcomes and health care utilizations compared to those living in neighbourhoods with low immigration scores. Although we cannot disentangle the individual versus the area-based effect of immigration, these results may have an important impact for health care planning.

**Electronic supplementary material:**

The online version of this article (doi:10.1186/s12913-015-0824-1) contains supplementary material, which is available to authorized users.

## Background

With a population health perspective, optimal allocation of resources requires understanding neighbourhoods’ health status and health care utilization. While the association of neighbourhood deprivation with poor health and disparities in health care utilization is increasingly recognized [1,2], few studies have taken an interest in the effects of neighbourhood immigration on health status [3,4]. Moreover, to our knowledge, none have explored the potential effects of neighbourhood immigration on health care utilization, even though immigrants represented about 21% of the Canadian population in 2011 [5].

The Andersen behavioural model [6] classifies the determinants of health care utilization according to three categories: predisposing (e.g., demographic and social factors, beliefs about health care, etc.), enabling (personal resources, availability of services within the community), and needs-related (current health status) factors. As a predisposing factor of health care utilization, studies on immigration (defined at the individual level) have provided mixed results. Some have reported significant differences in health care utilization between immigrants and the general population [7-11] while others did not find significant differences [12,13]. More specifically, some studies have reported that immigrants generally use fewer primary health care services [8,11,14], whereas other studies reached opposite conclusions [15]. It has been reported that immigrants generally have more visits to emergency departments and to specialists [16-19].

In short, the effect of immigration (either individually or by geographic region) on health care utilization and health outcomes is overall unclear. Two conflicting forces oppose each other when the health of immigrants is considered. On the one hand, immigrants tend to have a lower socioeconomic status (SES) than the majority of the population, which is known to be strongly associated with poor health and mortality [20-22]. The reduced recognition of diplomas, work skills, and work experience acquired abroad as well as other causes of lowered SES increase stress and affect mental health, causing a deterioration of physical health which contributes to the decline of general health of immigrants over time [23]. On the other hand, there is a well-known “healthy immigrant effect”, where immigrants are generally in better health [24-38]. Two processes are proposed to explain this healthy immigrant effect: a) a correlation between the capacity and desire to emigrate, and health; and b) a selection by receiving countries of “fittest immigrants” based on education level, language knowledge and professional skills, characteristics that facilitate social and economic integration and are associated with health [39]. These selection processes apply only to the so-called “economic immigrants”, but not to immigrants received for family reunion or as refugees [40]. However, “economic immigrants” represent a majority (about 62%) of immigrants in Canada.

Despite their overall “better health”, some immigrant populations are known to be at particularly high risk of diabetes compared to the general population. Recent findings suggest that populations of specific ethnic groups develop diabetes and other metabolic disorders at a higher rate, at an earlier age and in a lower range of body mass index [41-45]. Secondary data analyses of the Canadian Community Health Survey (CCHS) have shown that immigrants are at higher risk of diabetes than Canadian-born citizens [7,46]; however, immigrants were healthier overall than Canadian-born citizens, with an important reduction in the risk of having at least one physical or one mental health condition. Immigrants with diabetes were also less likely to have another chronic condition in addition to diabetes [47]. The most likely explanations reported for this high risk of diabetes involved “interactions between physiological predisposition characteristics of certain ethnocultural or country of origin groups, lack of exercise and the adoption of unhealthy diets” [7,48,49].

From the behavioural model [6] perspective, immigration is a predisposing factor for health care utilization. Since poorly managed diabetes may lead to serious complications and death, it is important to evaluate the effect of enabling factors on health care utilization and outcomes, a prime example being the immigration attributes of the neighbourhood. There is a public health interest in better understanding the variation of health care needs according to the concentrations of immigrants in different neighbourhoods (“neighbourhood immigration”).

The goal of this study is thus to explore how neighbourhood immigration may affect health outcomes and health care utilization for individuals living with diabetes. More precisely, for a population of patients with diabetes without cardiovascular disease (CVD) living in the Montreal metropolitan area in 2007, this study will measure: 1) the association between neighbourhood immigration and major health outcomes such as all-cause death, all-cause hospitalization, and cardiovascular events; 2) the association between neighbourhood immigration and the use of antidiabetic drugs as well as the frequency of emergency department (ED), general practitioner (GP), and specialist physician visits.

## Methods

### Study setting

Quebec receives more than 45,000 [50] of the 250,000 [51] new immigrants arriving in Canada each year. As most immigrants in Canada (92%) live in metropolitan areas, our study takes place in the most populated metropolitan area (i.e. Montreal) of the Province of Quebec in Canada [52]. In 2006, 11.5% of Quebec’s population was immigrant, and the great majority was concentrated in the metropolitan region of Montreal. In fact, almost a third of the Montreal population were immigrants compared to only 3% in other regions [53]. The origins of immigrants in Quebec (e.g. Africa and the Middle-East, Europe, South and Central America) are different than in the rest of Canada (e.g. Asia and Pacific) mainly because of the French culture concentration in Quebec with predominantly French-speaking people.

### Design and data sources

This is a population-based retrospective cohort study. Patient data were obtained from the provincial health insurance board (*Régie de l’assurance maladie du Québec*: RAMQ), which provides universal health insurance to Quebec residents, including coverage for physician and hospital services as well as a provincial drug insurance plan [54]. The RAMQ owns and manages administrative health registers including the hospital discharge (MED-ECHO), patients’ demographic information, physicians’ reimbursement claims for health care (including hospital inpatients and outpatients, emergency and private clinics visits), and drug reimbursement claims for eligible patients. The data are routinely collected for billing purposes by physicians, hospitals, and pharmacies, and to monitor population health. The MED-ECHO registry contains information on dates of hospitalizations, length of stay and the main diagnosis and up to 29 secondary diagnoses using the ICD-9 coding system before 2006 and ICD-10 thereafter. The RAMQ demographic database provides information on patients’ age, gender, and eligibility to the public drug insurance plan. The physician reimbursement claims register provides the date of service and the diagnosis (according to ICD-9) specific to the medical visit, while the drug register contains information on the drugs claimed by any individual covered by the public drug insurance plan. The mortality register provided by the *Institut de la statistique du Québec* (ISQ) includes the date and the cause of death (as coded using ICD-10) [55]. Using a unique encrypted identifier, patient data from all these registers are linked to provide information on demographic characteristics, medical and drug information as well as the postal code at the patient level.

Neighbourhood attributes are measured using the available information for dissemination areas (DA) in the Census Metropolitan Area (CMA) of Montreal as provided by the 2006 Census of population (Statistics Canada 2006). Each patient was spatially linked to one and only one DA using the postal code conversion file (PCCF) from Statistics Canada [56] to match the postal code of residence. Spatial information came from the DA cartographic boundary files for the Census Metropolitan Area (CMA) of Montreal of the 2006 Census of population [57]. The follow-up period was 24 months for every patient.

This study was approved by the Research Ethics Board Committee of the University of Sherbrooke and by the *Commission d’accès à l’information* of Quebec.

### Case definition of diabetes

The most common diabetes case definition is the one used by Health Canada in the National Diabetes Surveillance System (NDSS) and includes one hospitalization or two outpatient visits for diabetes over a 2-year period [58]. Different diabetes case definitions have been validated by Hux et al. [59], including the NDSS one. The NDSS algorithm had a sensitivity of 86% and a positive predictive value of 80%. Because of confidentiality issues, only a subset of the population meeting the NDSS inclusion criteria was available. In fact, a patient was considered living with diabetes if he/she has received a primary or secondary diagnosis of diabetes (ICD-9: 250; ICD-10: E10-E14) during a hospitalization or at least three physician claims with a diagnosis of diabetes (ICD-9: 250) within one year. Although this algorithm has not been explicitly validated, we can expect that the selected algorithm for this study will have a low sensitivity but a very high specificity.

### Studied population

The studied population included all individuals 30 years and older living in the CMA of Montreal and identified as having diabetes (according to the case definition described above) between January 2004 and December 2007. The first diagnosis observed during the study period will be used as the reference date. To investigate patients without a history of cardiovascular diseases, as well as women with possible gestational diabetes, those with a diagnosis of CVD (ICD-9: 410–414, 428, 430–438; ICD-10: I20-I25, I50, I60-I69) in the four years preceding the reference date or an obstetrical event (ICD-9: 630–676, 760–779, V22-V24, V27-V28; ICD-10: O00–O99, Z32-Z39) in the 5 months following the reference date [59] were excluded. Finally, patients living in DAs with missing socioeconomic data were also excluded (<5%).

**Figure 1:**
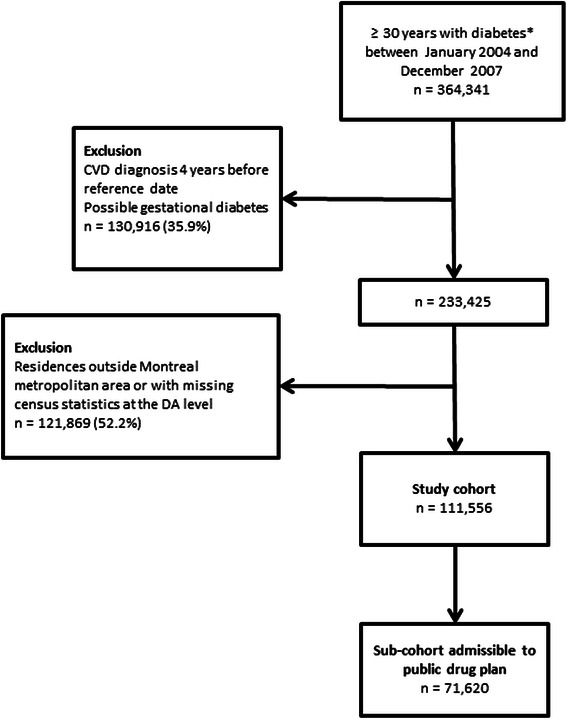
**Study cohort.** *Diagnosis of diabetes during hospitalization or 3 physician claims within one year with a diagnosis of diabetes.

The study cohort was thus composed of 111,556 patients living with diabetes, 30 years and older, without previous antecedent of CVD, and living in the metropolitan area of Montreal between January 2004 and December 2007 (Figure 1). Only patients covered by the public drug insurance plan were retained for the analyses on drug utilization, which represented 64% of the total study cohort (n = 71,620).

### Dependent variables: health indicators

At the patient level, the following binary variables were defined in a **2-year** period after the reference date: all-cause death; all-cause hospitalization; CVD event as defined by either CVD death or CVD hospitalization (ICD-9: 410–414, 428, 430–438; ICD-10: I20-I25, I50, I60-I69); frequent use of ED care (at least 4 visits at EDs); frequent use of GP care (at least 20 visits at a GP in an ambulatory care setting); frequent use of specialists care (at least 5 visits at a MD specialist in an ambulatory setting); and the purchase of at least one antidiabetic drug (any class among biguanides, sulfonylureas, insulin, thiazolidinediones). The thresholds used to categorize the use of care as “frequent” correspond to the third quartile of care utilization (i.e. 75% of the study cohort had a lower utilization). Naessens et al. [60] used the same threshold fixed at 20 visits for a 2-year follow-up period to determine the frequent users in primary care.

### Independent variables: individual and neighbourhood-level measures

#### Patient-level variables

The following variables were selected at the patient level: sex, age at reference date, being an incident case of diabetes [61] (defined as the first diagnosis of diabetes over a 4-year period before the reference date), having diabetes-related complications, and having comorbidities, namely, hypertension, dyslipidemia, dementia, chronic pulmonary disease, renal disease, connective tissue disease, ulcer disease, mild liver disease, moderate to severe liver disease, any tumor, leukemia, lymphoma, or metastatic tumor. Most of these comorbidities are those derived from the Charlson comorbidity index (as adapted for the RAMQ database by D’Hoore et al.) [62,63]. A patient was considered to have one of these conditions if he/she received at least one diagnosis (main or secondary) for this condition within four years prior to the reference date (see Additional file 1: Table S1 for a list of ICD-9 and ICD-10 diagnostic codes for each selected comorbidity).

#### Geographical-level (DAs) variables

**Figure 2:**
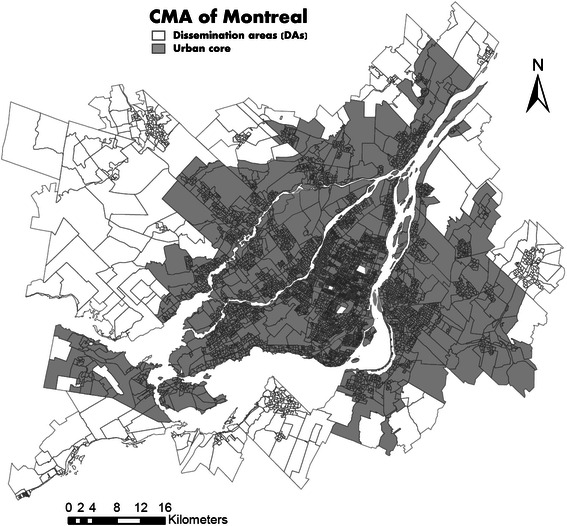
**Map of dissemination areas (DAs) of the census metropolitan area (CMA) of Montreal and the urban core delimitation.**

Since the availability of some health services may differ within the metropolitan area (e.g., concentration of specialists and emergency departments in the city core), a binary variable was defined to indicate if the DA is located in the urban core of the CMA of Montreal. Figure 2 gives a geographic representation of the DAs of the CMA of Montreal and the delimitation of the urban core. As defined by Statistics Canada, an “urban core” is a large urban area around which a CMA is delineated. The urban core must have a population of at least 50,000 persons (based on the previous census), and includes both central business districts and peripheral residential neighbourhoods [64]. Using the PCCF [56], each DA was classified as being located or not in the urban core.

### Other DA-level variables: immigration, material and social indices

Social and material deprivation and other area-based socioeconomic indices are used extensively in public health studies [65-70]. A validated [71] index widely used in Quebec and Canada [72] proposed a two-dimensional deprivation index (material and social). However, this index does not take into account variables related to spoken languages and the proportion of recent and previous immigrants. Some other studies have considered neighbourhood immigration attributes [4,73]. An index developed in Italy included five dimensions of deprivation, including immigration, to study the role of socioeconomic factors on the prevalence of diabetes and associated adverse outcomes but, as this was done to create a unique composite index of deprivation, it was impossible to disentangle the specific role of immigration. We thus propose here to create a specific immigration index.

Statistics Canada [74] defines “immigrants” as: “people who are, or have ever been, landed immigrants in Canada, which means people who have been granted the right to live in Canada permanently by immigration authorities”. Some immigrants have resided in Canada for many years, while others have recently arrived. Hence, immigrants do not include non-permanent residents (people from another country who had a Work or Study Permit, or who were refugee claimants at the time of the census, and family members living in Canada with them) [75].

Many Canadian census variables are related to immigration and/or spoken languages that may be used for the development of an immigration index. However, since several of them are strongly correlated (e.g. “Proportion of people who do not speak French or English” and “Place of birth”) while others refer to long-term immigrants (“Proportion of immigrants that arrived more than 25 years ago in Canada”), we selected the following variables: 1) The proportion of allophones in the population, defined as people for whom the maternal language is neither French nor English, the two official languages in Canada; 2) The proportion of population who do not speak French or English; 3) The proportion of recent immigrants (arrived in Canada 5 years ago or less); 4) the proportion of immigrants that arrived between 5 and 10 years ago in Canada; 5) The proportion of immigrants that arrived between 10 and 15 years ago in Canada; and 6) the proportion of immigrants that arrived between 15 and 25 years ago in Canada.

The selection of socioeconomic variables was based on the deprivation indices developed in Quebec [71,72]. The material deprivation index was calculated using the employment rate, the average income of people 15 years and older, and the proportion of the population who do not have a high school certificate. The social deprivation index was calculated using the proportion of the population living alone, the proportion of the population separated, divorced, or widowed, and the proportion of single-parent families. All these variables were available at the DA-level from Statistics Canada for the 2006 population census.

Principal component analysis (PCA) [76] was used to convert the set of correlated variables into a reduced set of uncorrelated variables. This orthogonal transformation is defined in such a way that the first principal component (or axis) has the largest possible variance, that is, accounts for as much of the variability in the data as possible (see the statistical analyses section for more details). In this study, each dimension was analysed separately. For half of the variables, particularly those related to immigration, it was not possible to obtain estimates by age group and sex for each DA, so the census variables were not adjusted for age and sex. Rather, adjustment was done at the modeling level (see below).

### Statistical analyses

First, immigration, material, and social dimension scores were calculated for all DAs of the CMA of Montreal using their associated census variables. These variables were transformed (log, square or square root transformations) if needed to reach normality and then standardized. Three separated PCA analyses (the FACTOR procedure, SAS 9.2) were performed for each group of variables related to immigration, material and social dimensions. Only the most important axis (i.e., that with the highest eigenvalue) was extracted for each dimension. For each axis, DAs were sorted according to the ascending PCA scores and grouped in quintiles of population [77-79].

For all dependent variables (health care utilization and outcomes) and their associations with immigration and deprivation indices, adjusted odds ratios (OR) were estimated using a multilevel logistic regression (GLIMMIX procedure in SAS 9.2 [80]). Multilevel regressions were used as they appropriately model each health indicator with patient-level and DA-level explanatory variables. All ORs were adjusted for all patient-level variables (age, sex, being an incident diabetes case, comorbidities, etc.) and whether or not DAs were from the urban core. Eq.1 presents the general underlying multilevel equation used in this study.

1 $$ logit\left({\pi}_{ij}\right)=\alpha +{u}_j+\boldsymbol{\gamma} {\boldsymbol{z}}_j^t+\boldsymbol{\beta} {\boldsymbol{x}}_{ij}^t $$

where *π*_*ij*_ is the probability of the dependent variable (e.g. death) for patient *i* living in neighbourhood (DA) *j*, *u*_*j*_ is the DA random effect, ***z***^*t*^_*j*_ is the (transpose) vector of DA-level variables and ***x***^*t*^_*ij*_ is the (transpose) vector of patient-level variables, *α* is the model intercept, ***γ*** is the vector of coefficients for DA-level covariates, and ***β*** is the vector of coefficients for individual level covariates.

To test if results were sensitive to the definition of immigration and deprivation indices, regression models differed in the way the DA-level variables (immigration, material deprivation, and social deprivation scores) were included in the general multilevel model. Some models included the immigration, material deprivation, and social deprivation dimensions as three continuous variables while others included them as quintiles. In order to see how immigration and deprivation interact, we combined immigration quintiles with material deprivation quintiles (25 categories) and combined immigration quintiles with social deprivation quintiles (25 categories). The intraclass correlation (ICC) [81] and median odds ratio (MOR) [82] are also presented alongside multilevel regression models. The ICC gives the proportion of total variance in the outcome that is attributable to the DA-level, and the MOR gives the median value of the odds ratio between the DA at higher risk and the DA at lower risk when randomly picking out two DAs. This facilitates interpretation of ORs for continuous variables, where the effect is per unit and is exponential. Mappings were done using ArcGIS 10.2, and all statistical analyses were performed using SAS 9.2 software package.

#### Sensitivity analyses

First, different PCA analyses were performed and their scores compared with those of the original model (correlation coefficients) to test the effect of the proposed immigration, material, and social indices on the selected health indicators. Second, to see if the results were sensitive to the way we defined health care utilization, we considered other dependent variables such as the number of health care utilizations (EDs, GPs, and specialists). These variables were analysed using three different multilevel models: Poisson, negative-binomial, and zero-inflated models. Third, to overcome the potential survival bias that can occur if differences of death rates occur between immigrants and Canadian-born citizens, we performed analyses that included only patients that survived the 2-year follow-up period. Finally, we performed separated analyses for incident and prevalent diabetes cases.

## Results

### Immigration, material and social dimensions

PCA analyses produced only one major axis for each of the three dimensions that explained more than 60% of the variation (Table 1). Each axis is a linear combination of the selected census variables related to its respective dimension (immigration, material deprivation, and social deprivation). For each DA, immigration, material, and social scores were calculated and grouped in population quintiles.

**Table 1:** **Component loadings of the 12 census variables (n = 6,009 DAs)**

	Dimension		
Axes (derived from 3 PCAs)	Immigration	Material	Social
Eigenvalue	3.73	1.91	1.87
Percentage of variance explained by the axis	62.2	63.5	62.3
Eigenvalue of the second most important axis	0.64	0.65	0.76
**Component loadings associated with variables** ^**1**^			
Proportion of people with maternal language other than French or English**	0.92	−	−
Proportion of people who does not speak French or English**	0.79	−	−
Proportion of recent immigrants (arrived < 5 years ago in Canada)**	0.75	−	−
Proportion of immigrants that arrived 5-10 years ago**	0.73	−	−
Proportion of immigrants that arrived 10-15 years ago**	0.77	−	−
Proportion of immigrants that arrived 15-25 years ago**	0.75	−	−
Average income**	−	-0.82	−
Employment rate***	−	-0.73	−
Proportion of people without high school certificate*	−	0.84	−
Proportion of separated, divorced or widowed people*	−	−	0.86
Proportion of people living alone**	−	−	0.86
Proportion of single-parental families**	−	−	0.63

*Logarithmic transformation **Square root transformation ***Square transformation.

^1^Component loadings (corresponding to correlations between the component and the variables) associated with census variables after transformation and standardization, in that order.

**Figure 3:**
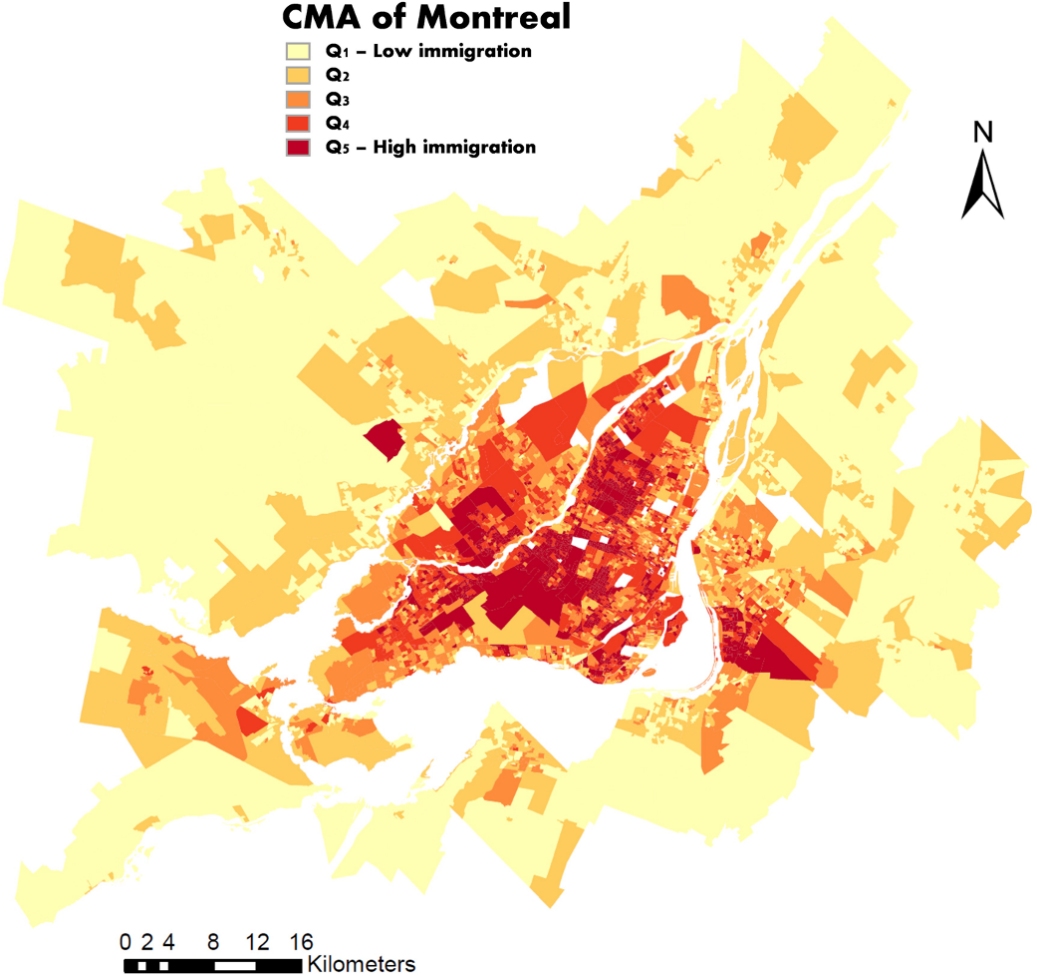
**Map of the immigration quintiles in the CMA of Montreal.**

**Figure 4:**
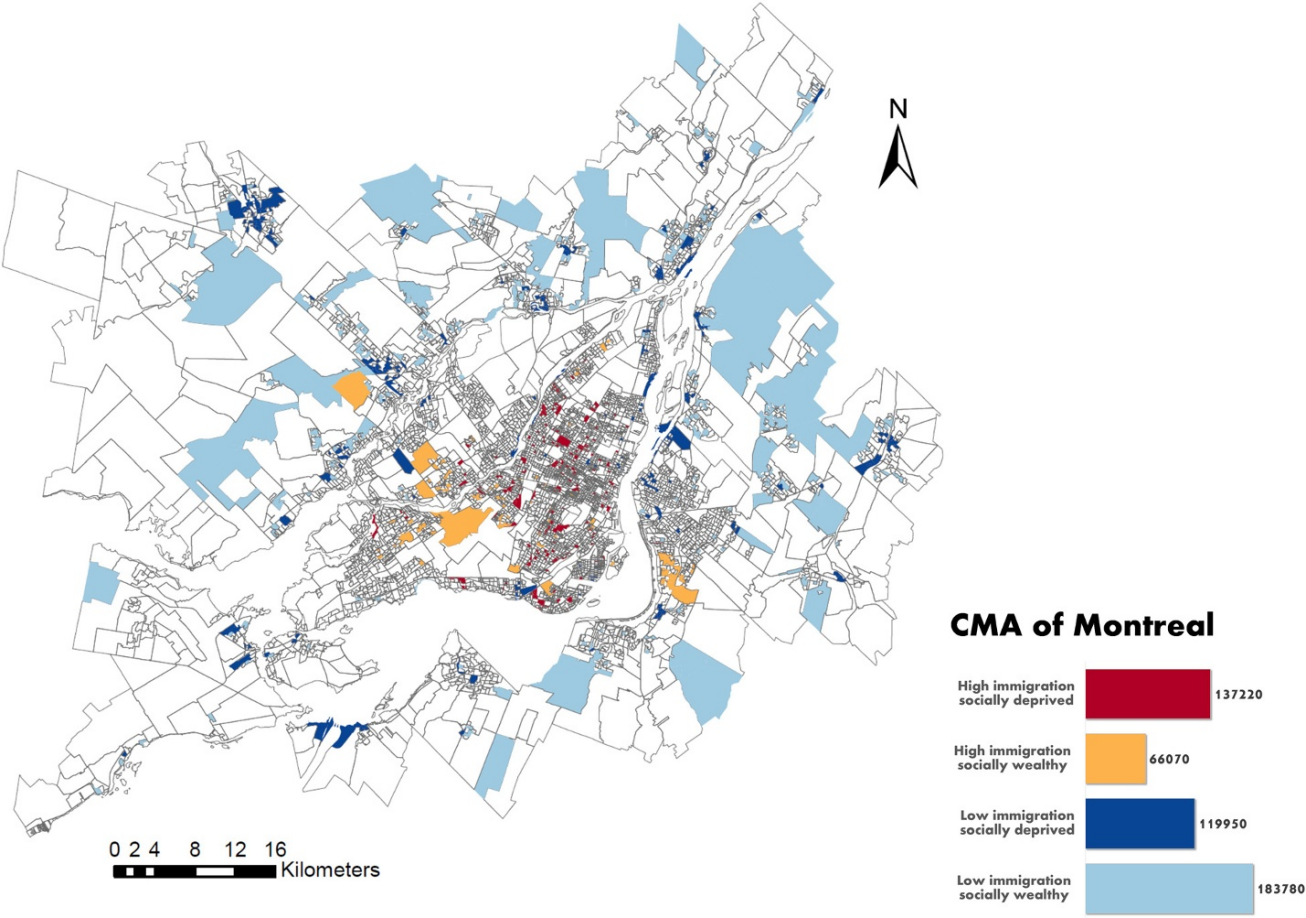
**Map of the immigration and social deprivation subpopulations in the CMA of Montreal.**

**Figure 5:**
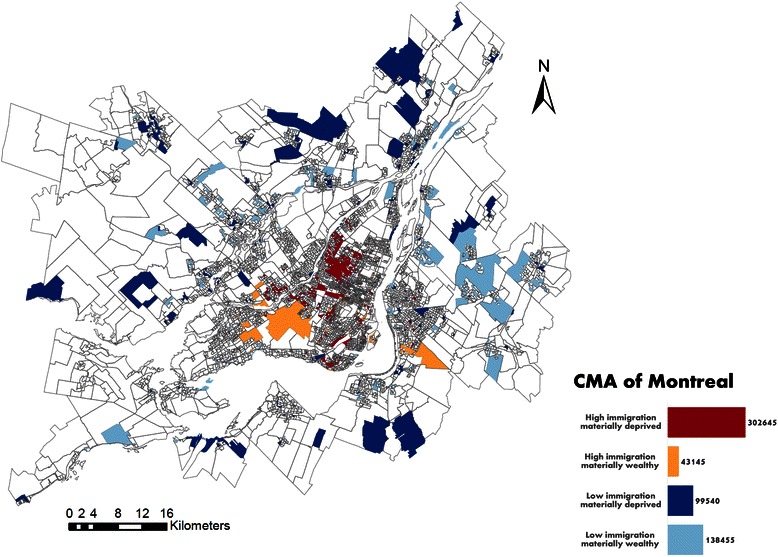
**Map of the immigration and material deprivation subpopulations in the CMA of Montreal.**

Immigrant subpopulations settled predominantly in the urban core while Canadian-born subpopulations settled predominantly in the suburban areas (Figures 3, 4 and 5). Only 40 of the 6,009 DAs (for a total population of 43,145 individuals) were at the same time in the “high immigration” quintile and in the “materially wealthy” quintile (Table 2 and Figure 5). These neighbourhoods are mostly concentrated in the west part of the Montreal Island, whereas the “high immigration materially deprived” neighbourhoods are clustered in the North-Eastern part.

**Table 2:** **Characteristics (mean and standard deviation) of the CMA of Montreal** ^**1**^ **: DA-level variables**

	CMA of Montreal	Socially wealthy (Q1)		Socially deprived (Q5)		Materially wealthy (Q1)		Materially deprived (Q5)	
		Low immigration (Q1)	High immigration (Q5)	Low immigration (Q1)	High immigration (Q5)	Low immigration (Q1)	High immigration (Q5)	Low immigration (Q1)	High immigration (Q5)
Number of DAs	6,009	339	95	214	200	230	40	184	502
Total population	3,606,770	183,780	66,070	119,950	137,220	138,455	43,145	99,540	302,645
Immigration score^2^	0 (3.7)	−4.5 (0.5)	5.1 (1.4)	−4.5 (0.5)	5.3 (1.5)	−4.4 (0.5)	4.6 (1.0)	−4.6 (0.5)	6.3 (1.9)
Social deprivation score^2^	0 (1.9)	−2.5 (0.7)	−2.6 (0.8)	2.8 (1.1)	2.3 (0.7)	−1.8 (1.3)	−1.2 (1.6)	1.7 (2.1)	0.7 (1.3)
Material deprivation score^2^	0 (1.9)	−1.3 (1.5)	−0.5 (1.8)	1.6 (1.5)	1.7 (1.5)	−2.5 (0.7)	−2.4 (0.7)	2.5 (0.9)	2.7 (0.8)
**DA-level variables** ^**3**^									
% of people with maternal language other than French or English	21.5 (19.5)	3.3 (2.6)	49.6 (11.6)	3.3 (2.3)	42.4 (11.4)	3.6 (2.8)	39.7 (8.4)	3.1 (2.6)	55.8 (14.8)
% of people who do not speak French or English	1.6 (2.8)	0.02 (0.2)	4.9 (3.9)	0.03 (0.18)	4.2 (3.6)	0.01 (0.14)	2.9 (1.3)	0.04 (0.25)	6.5 (4.6)
% of recent immigrants^*^ (<5 years)	4.5 (6.6)	0.2 (6.6)	5.8 (7.5)	0.3 (0.8)	13.0 (7.4)	0.2 (0.7)	6.8 (6.4)	0.2 (0.6)	13.4 (9.1)
% of immigrants^*^ (5–10 years)	2.5 (3.6)	0.1 (0.5)	5.5 (4.2)	0.1 (0.4)	7.0 (5.1)	0.2 (0.5)	5.5 (3.4)	0.1 (0.4)	6.8 (4.9)
% of immigrants^*^ (10–15 years)	2.6 (3.6)	0.1 (4.6)	6.8 (5.2)	0.1 (0.4)	6.7 (4.2)	0.1 (0.5)	5.9 (3.0)	0.1 (0.4)	7.6 (4.5)
% of immigrants^*^ (15–25 years)	3.4 (4.0)	0.2 (6.7)	9.2 (3.9)	0.2 (0.5)	6.9 (4.3)	0.2 (0.6)	8.9 (3.7)	0.2 (0.6)	8.2 (4.7)
Average income (x 1000)	34.3 (16.2)	40.2 (11.3)	37.6 (15.1)	27.7 (6.2)	23.6 (6.0)	46.1 (11.6)	53.2 (13.9)	25.2 (5.2)	20.4 (3.9)
Employment rate	62.2 (12.0)	73.2 (8.6)	63.7 (9.9)	53.6 (15.7)	50.0 (9.2)	77.4 (7.5)	69.8 (5.9)	49.9 (13.9)	48.2 (7.7)
% of people without high school certificate	22.0 (10.7)	19.4 (9.0)	18.7 (10.0)	31.9 (9.1)	26.1 (12.8)	12.6 (4.4)	10.7 (4.1)	38.9 (7.3)	35.0 (8.6)
% of separated, divorced or widowed people	18.3 (6.8)	12.4 (2.4)	10.4 (2.2)	30.6 (7.9)	24.6 (4.8)	14.2 (4.2)	14.0 (4.6)	27.2 (10.2)	28.9 (4.7)
% of people living alone	13.4 (10.1)	3.4 (2.2)	4.6 (5.0)	23.8 (10.1)	23.7 (8.6)	4.8 (4.0)	10.6 (8.2)	19.8 (12.5)	14.2 (7.2)
% of single-parental families	18.1 (10.7)	7.7 (5.4)	8.3 (5.6)	26.0 (11.4)	31.2 (12.2)	10.9 (7.0)	10.9 (6.8)	21.0 (12.7)	27.2 (11.3)

^*^Among the total population.

^1^Results from the 2006 Canadian census of population

^2^Immigration score as well as Social and Material scores are obtained from principle component analyses (see the Methods section and Table 1).

^3^Mean and standard deviation (weighted by the DA population).

As expected, characteristics associated with immigration are very high in neighbourhoods with high immigration (quintile 5 of the immigration axis) as compared to neighbourhoods with low immigration (quintile 1 of the immigration axis) (Table 2). For example, in neighbourhoods with low immigration, the average proportion of immigrants that arrived less than 25 years ago was lower than 1% while in neighbourhoods with high immigration it was higher than 28%.

Additional PCA analyses were performed pooling all selected variables (with and without adjustment for age and sex when possible) and then the three most important principal components were retained. Since the components loadings depend on the rotation method used, we performed both oblique and orthogonal rotations. The oblique rotation is often used when factors are believed to be correlated. These four PCA analyses produced an immigration axis with similar interpretation (data not shown) to the one retained in this paper (Table 1) and the correlation coefficients between the various scores were almost perfect and exceeded 0.99. For the material and social axes, the comparisons were not so clear because the axes did not always split variables in material and social dimensions, but were sometimes a combination of the census variables related to these dimensions. This indicates that immigration is a more distinct, independent phenomenon than material and social deprivation.

### Health indicators of the study cohort

The characteristics of the study cohort are presented in Table 3. The cohort was composed of 48.7% women, 51.3% men and the average age was 61.3 years old. Regarding patients’ characteristics such as age, sex, and comorbidities, statistically significant differences by quintiles of immigration were noted (Table 3), with negative trends for most comorbidity diagnoses. Patients with diabetes living in neighbourhoods with high immigration (Immigration - Q5) were mostly concentrated in the urban core (95.9%) as compared to neighbourhoods with low immigration (65.0%) (Table 3). There is an uneven distribution of the study cohort across immigration quintiles, as well as across social and material quintiles. Patients living in neighbourhoods with high immigration were also more likely to live in neighborhoods with high material deprivation and, to a lesser extent, with high social deprivation (Table 3).

**Table 3:** **Description of the study cohort living in the CMA of Montreal (n = 111,556): patient- and DA-level variables by immigration quintiles**

				Immigration			Trend test**
Variables	TOTAL	Q1 - Low	Q2	Q3	Q4	Q5 - High	β (p - value)
TOTAL study cohort, n (%)	111,556 (100)	19,833 (17.8)	20,838 (18.7)	22,240 (19.9)	23,318 (20.9)	25,327 (22.7)	−
Patient-level variables							
Average age (SD)	61.3 (13.1)	60.7 (12.9)	60.9 (13.0)	61.8 (13.1)	61.9 (13.3)	61.2 (13.3)	0.184 (<.0001)
Sex, n (%)							
Women	54,305 (48.7)	9,266 (46.7)	9,853 (47.3)	10,847 (48.8)	11,581 (49.7)	12,758 (50.4)	0.039 (<.0001)
Men	57,251 (51.3)	10,567 (53.3)	10,985 (52.7)	11,393 (51.2)	11,737 (50.3)	12,569 (49.6)	−
Incident diabetes cases, n (%)	30,621 (27.4)	5,286 (26.6)	5,742 (27.6)	6,226 (28.0)	6,520 (28.0)	6,847 (27.0)	0.005 (0.3353)
Diabetes with complications, n (%)	10,286 (9.2)	1,740 (8.8)	1,923 (9.2)	2,093 (9.4)	2,213 (9.5)	2,317 (9.2)	0.011 (0.1393)
Hypertension, n (%)	19,267 (17.3)	3,364 (17.0)	3,674 (17.6)	3,958 (17.8)	4,092 (17.6)	4,179 (16.5)	−0.009 (0.1193)
Dyslipidemia, n (%)	8,821 (7.9)	19,833 (8.2)	1,795 (8.6)	1,803 (8.1)	1,786 (7.7)	1,801 (7.1)	−0.046 (<.0001)
Dementia, n (%)	1,977 (1.8)	362 (1.8)	401 (1.9)	421 (1.9)	407 (1.8)	386 (1.5)	−0.047 (0.0031)
Chronic pulmonary disease, n (%)	4,153 (3.7)	775 (3.9)	835 (4.0)	882 (4.0)	827 (3.6)	834 (3.3)	−0.049 (<.0001)
Renal disease, n (%)	4,136 (3.7)	828 (4.2)	827 (4.0)	860 (3.9)	779 (3.3)	842 (3.3)	−0.065 (<.0001)
Connective tissue disease or ulcer disease, n (%)	1,308 (1.2)	216 (1.1)	224 (1.1)	262 (1.2)	252 (1.1)	354 (1.4)	0.057 (0.0041)
Liver disease, n (%)	2,433 (2.2)	442 (2.2)	488 (2.3)	495 (2.2)	500 (2.1)	508 (2.0)	−0.031 (0.0300)
Tumor (incl. leukemia, lymphoma, metastatic tumor), n (%)	5,510 (4.9)	1,009 (5.1)	1,065 (5.1)	1,172 (5.3)	1,112 (4.8)	1,152 (4.6)	−0.032 (0.0012)
Neighbourhood (DA)-level variables							
Living in the urban core, n (%)	96,862 (86.8)	12,889 (65.0)	17,046 (81.8)	20,501 (92,2)	22,146 (95.0)	24,280 (95.9)	0.722 (<.0001)
Average material deprivation score (SD)	0.52 (1.82)	0.33 (1.66)	0.06 (1.78)	0.08 (1.84)	0.41 (1.75)	1.51 (1.60)	0.290 (<.0001)
Average social deprivation score (SD)	0.33 (1.89)	0.04 (2.04)	0.23 (2.08)	0.48 (2.03)	0.45 (1.86)	0.42 (1.44)	0.093 (<.0001)

**Linear regressions (continuous variables) or logistic regressions (binary variables) were used to model trends (+: increasing, −: decreasing) for each observed variables (first column) over the quintiles of immigration.

**Figure 6:**
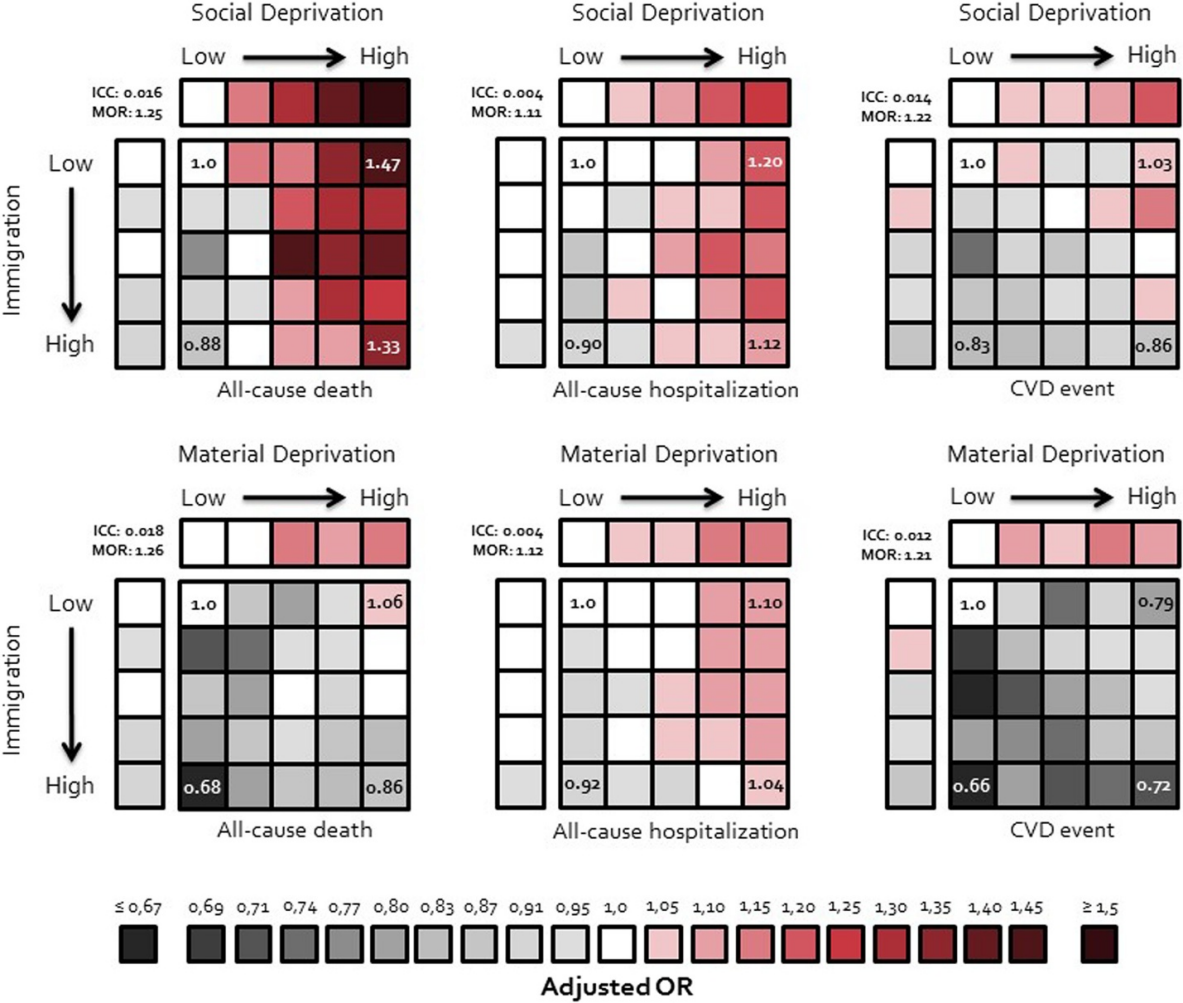
**Outcomes adjusted odds ratios (OR) associated with immigration by deprivation defined neighbourhoods as compared to low immigration wealthy neighbourhoods (reference group): multilevel logistic regression models.** *This is the representation of six multilevel logistic regression models. The dependent variable is indicated at the bottom of each square. The independent categorical variable is the combination of immigration quintiles with social deprivation quintiles (3 top squares) or with material deprivation (3 bottom squares). The reference category refers to neighbourhoods in the first immigration quintile and the first deprivation quintile (low immigration and less deprive neighbourhoods). All models were adjusted for age, sex, being an incident or prevalent diabetes case, having diabetes with complications, presence of comorbidities (hypertension, dyslipidemia, dementia, chronic pulmonary disease, renal disease, connective tissue disease, ulcer disease, mild liver disease, moderate to severe liver disease, any tumor, leukemia, lymphoma, metastatic tumor), and living in the urban core. ICC: intraclass correlation. MOR: median odds ratio.

After a 2-year follow-up, the cohort accumulated 6,453 (5.8%) deaths, 35,928 (32.2%) hospitalizations and 6,064 (5.4%) CVD events (Table 4). Except for all-cause hospitalization, these outcome events were generally less likely for patients living in neighbourhoods with high immigration than those living in neighbourhoods with low immigration (Table 4) and this remained even after controlling for patient-level variables (Table 5, Figure 6) such as age, sex, being an incident diabetes case, comorbidities and living in the urban core.

**Table 4:** **Description of the study cohort living in the CMA of Montreal (n = 111,556): outcomes and health care utilizations by immigration quintiles**

				Immigration			Trend test **
Dependent variables	TOTAL	Q1 - Low	Q2	Q3	Q4	Q5 - High	β (p - value)
TOTAL study cohort, n (%)	111,556 (100)	19,833 (17.8)	20,838 (18.7)	22,240 (19.9)	23,318 (20.9)	25,327 (22.7)	−
All-cause death, n (%)	6,453 (5.8)	1,172 (5.9)	1,205 (5.8)	1,364 (6.1)	1,352 (5.8)	1,360 (5.4)	−0.021 (0.0203)
All-cause hospitalization, n (%)	35,928 (32.2)	6,370 (32.1)	6,711 (32.2)	7,304 (32.8)	7,605 (32.6)	7,938 (31.3)	−0.006 (0.1635)
CVD event, n (%)	6,064 (5.4)	1,109 (5.6)	1,216 (5.8)	1,201 (5.4)	1,299 (5,6)	1,239 (4.9)	−0.034 (0.0003)
ED frequent users (≥4), n (%)	29,247 (26.2)	4,617 (23.3)	5,049 (24.2)	5,844 (26.6)	6,443 (27.6)	7,294 (28.8)	0.075 (<.0001)
GP frequent users (≥20), n (%)	29,568 (26.5)	5,669 (28.6)	5,572 (26.7)	5,894 (26.5)	6,139 (26.3)	6,294 (24.8)	−0.040 (<.0001)
MD specialists frequent users (≥5), n (%)	29,975 (26.9)	4,238 (21.4)	5,306 (25.5)	6,333 (28.5)	6,771 (29.0)	7,327 (28.9)	0.092 (<.0001)
Sub-cohort admissible to public drug plan, n (%)	71,620 (100)	11,169 (15.6)	12,058 (16.8)	13,894 (19.4)	15,669 (21.9)	18,830 (26.3)	−
Antidiabetic drugs, n (%)	61,395 (85.7)	9,462 (84.7)	10,247 (85.0)	11,749 (84.6)	13,429 (85.7)	16,508 (87.7)	0.059 (<.0001)

**Logistic regression (binary variables) were used to model trends (+: increasing, −: decreasing) for each dependent variable (first column) over the quintiles of immigration.

**Table 5:** **Odds ratios** ^**1**^ **as measures of effect of immigration (continuous vs quintiles) on health indicator adjusted (or not) for material and social deprivation: multilevel logistic regression models**

	Continuous immigration score		Immigration quintiles	
Health indicators	Not adjusted for material & social deprivation scores	Adjusted for material & social deprivation scores	Not adjusted for material & social deprivation quintiles	Adjusted for material & social deprivation quintiles
	OR (95% CI)^2^	OR (95% CI)^2^	OR (95% CI)^3^	OR (95% CI)^3^
All-cause deaths	0.88 (0.78 – 0.99)*	0.89 (0.80 – 1.00)	0.90 (0.81 – 0.99)*	0.87 (0.78 – 0.96)*
All-cause hospitalizations	0.96 (0.91 – 1.00)	0.92 (0.87 – 0.97)*	0.97 (0.92 – 1.01)	0.93 (0.89 – 0.98)*
CVD events	0.82 (0.74 – 0.91)***	0.79 (0.70 – 0.87)***	0.86 (0.78 – 0.94)**	0.84 (0.77 – 0.93)**
ED frequent users (≥4)	1.51 (1.43 – 1.60)***	1.43 (1.35 – 1.52)***	1.38 (1.31 – 1.45)***	1.28 (1.21 – 1.34)***
GP frequent users (≥20)	0.79 (0.74 – 0.83)***	0.69 (0.65 – 0.73)***	0.82 (0.78 – 0.86)***	0.75 (0.71 – 0.79)***
MD specialists frequent users (≥5)	1.50 (1.41 – 1.60)***	1.83 (1.73 – 1.94)***	1.49 (1.41 – 1.58)***	1.64 (1.55 – 1.73)***
Antidiabetic drugs	1.32 (1.20 – 1.43)***	1.20 (1.11 – 1.32)***	1.21 (1.12 – 1.31)***	1.12 (1.03 – 1.22)**

*p < 0.05; **p < 0.001; ***p < 0.0001;

^1^Adjusted for age, sex, being an incident or prevalent diabetes case, having diabetes with complications, presence of comorbidities (hypertension, dyslipidemia, dementia, chronic pulmonary disease, renal disease, connective tissue disease, ulcer disease, mild liver disease, moderate to severe liver disease, any tumor, leukemia, lymphoma, metastatic tumor), and living in the urban core.

^2^OR are calculated using the coefficient obtained in the logistic regression (β) with a continuous immigration score. The OR presented here compares the 97.5^th^ and the 2.5^th^ percentile (reference group) of the immigration score.

^3^OR are calculated using the coefficient obtained in the logistic regression (β) with a categorical immigration score (quintiles). The OR presented here compares the 5^th^ quintile and the 1^st^ quintile (reference group) of the immigration score.

**Figure 7:**
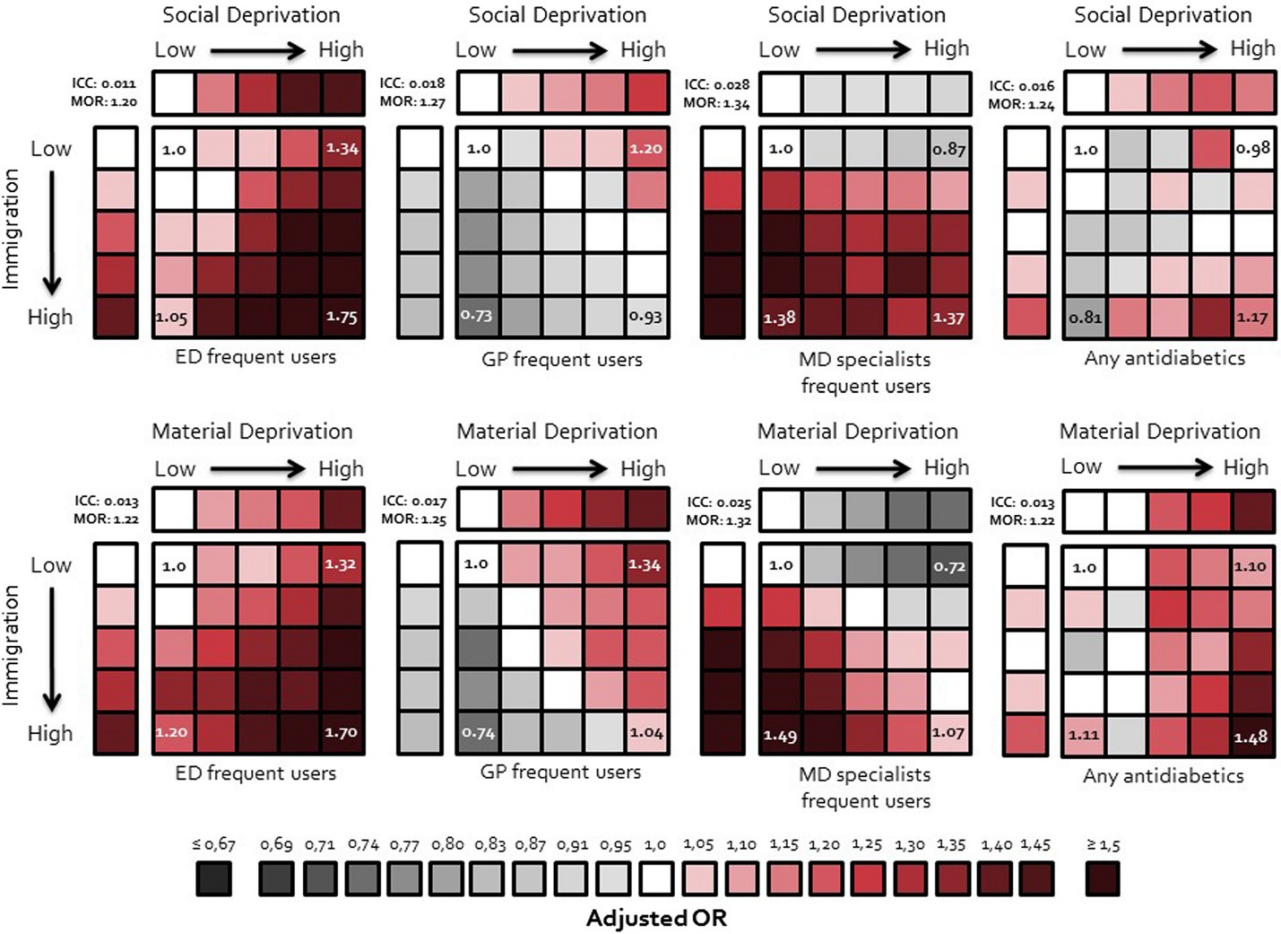
**Health care utilization adjusted odds ratios (OR) associated with immigration by deprivation-defined neighbourhoods as compared to low immigration wealthy neighbourhoods (reference group): multilevel logistic regression models.** *This is the representation of eight multilevel logistic regression models. The dependent variable is indicated at the bottom of each square. The independent categorical variable is the combination of immigration quintiles with social deprivation quintiles (4 top squares) or with material deprivation (4 bottom squares). The reference category refers to neighbourhoods in the first immigration quintile and the first deprivation quintile (low immigration and less deprive neighbourhoods). All models were adjusted for age, sex, being an incident or prevalent diabetes case, having diabetes with complications, presence of comorbidities (hypertension, dyslipidemia, dementia, chronic pulmonary disease, renal disease, connective tissue disease, ulcer disease, mild liver disease, moderate to severe liver disease, any tumor, leukemia, lymphoma, metastatic tumor), and living in the urban core. ICC: intraclass correlation. MOR: median odds ratio.

With respect to the utilization of health care services, 29,247 (26.2%) patients consulted EDs at least 4 times, 29,568 (26.5%) visited frequently (at least 20 times) GPs and 29,975 (26.9%) visited frequently (at least 5 times) MD specialists (Table 4), during the 2-year follow-up period. Adjusted results show that patients living in neighbourhoods with high immigration were more likely to consult at EDs and visit specialists than those living in neighbourhoods with low immigration (Table 5, Figure 7). Figure 7 shows very important and striking gradients in health care utilization: patients living in neighbourhoods with high immigration and deprivation (socially or materially) were the most important users of emergency care, while those living in neighbourhoods with high immigration and wealthy residents (socially or materially) had the highest rate of visit to specialists but the lowest rate of visit to a GP. Finally, socially or materially deprived neighbourhoods with a high immigrant score had the highest rate of utilization of an antidiabetic drug among those admissible to the public drug insurance plan (Figure 7). To see if the results were sensitive to the way we defined health care utilization, we analysed the number of health care utilizations (EDs, GPs, and MD specialists) using different multilevel models for count data (Poisson, negative-binomial, and zero-inflated models). All these nine models show a statistically significant increase of ED and MD specialist utilization but a statistically significant decrease of GP utilization for patients living in neighbourhoods with a high immigrant score as compared to those living in neighbourhoods with a low immigrant score.

Stratified analyses by incident and prevalent diabetes cases and analyses on a subpopulation of patients that survived the 2-year follow-up period give comparable results (data not shown).

## Discussion

The results reveal that, as compared to patients with diabetes living in neighbourhoods with a low immigration score, those living in neighbourhoods with a high immigration score were less likely to die, to suffer a CVD event, to frequently visit GPs, but more likely to frequently visit EDs and specialists and to use an antidiabetic drug. These differences remained even after controlling for patient-level variables such as age, sex, being an incident or prevalent diabetes case, comorbidities, as well as other neighbourhood attributes like material and social deprivation and living or not in the urban core.

Since immigration and socioeconomic variables are measured at the area-level, we cannot presume that the better outcomes and the difference in health care utilization observed in neighbourhoods with a high immigration score are actually attributable to immigrants. However, the lower risk of outcomes (death, CVD events) in neighbourhoods with a higher immigration score is in line with “the healthy immigrant effect”, where immigrants’ health (except for diabetes) is generally better than the majority of the population [10,24-38]. Another study also showed a reduced rate of death in newly diagnosed diabetes patients from Asia as compared to the others [83].

Regarding health care utilization, patients with diabetes living in neighbourhoods with a high immigration score were more likely to be frequent ED and MD specialists users but less likely to be frequent GP users than those living in neighbourhoods with a low immigration score. The significantly lower rate of frequent GP users observed in patients living in a neighbourhood with a high immigration score is in agreement with some studies [7,8], but not with others [10]. Beiser et al. [7] reported that recent immigrants with chronic health problems were less likely to have seen a family doctor than established immigrants (present in Canada for more than 10 years) or Canadian-born citizens with similar problems. Another study of immigrants in Italy [8] also reported immigrant underutilization of primary health care. The lower rate of visits to a GP combined with the higher use of EDs in neighbourhoods with high immigration can reflect a barrier to primary care access (e.g., difficulty to get an appointment) [14]. Also, it has been reported [17] that many immigrants did not consider health as a priority when they first arrived, so that little time was spent on getting acquainted with the health system in Quebec. Their first contact to the health care system was thus generally motivated by isolated health problems, where visits to EDs is often the only possible choice in country with general physician workforce shortages as in the province of Quebec [16]. Another Canadian study [18] reported a lower specialist waiting time for immigrants among male patients as compared to Canadian-born and a study conducted in the province of Quebec [19] reported a higher utilization rate of specialists in immigrant ethnic groups. Two situations associated with cultural or communication problems have been proposed to explain why patients living in neighbourhoods with high immigration scores consult more MD specialists than GPs [19]. One may be the difficulty for a GP to properly assess the health concerns of an immigrant patient and may tend to refer the patient more quickly to a specialist for further investigation and treatment. On the other hand, the patient may also ask to be referred if he or she does not feel well understood. Another possible explanation is that immigrant patients may have been referred to MD specialists by doctors seen at EDs.

Finally, materially deprived neighbourhoods with high immigration scores had the most important rate of antidiabetic drug utilization among those admissible to the public drug insurance plan. However, given the important rate of antidiabetic drug utilization (over 85%), the increase is moderate. It is possible that patients living in less deprived neighbourhoods are being diagnosed at an early stage of the disease (thus needing only behavioural treatment) when compared to patients in more deprived neighbourhoods. Another study on schizophrenia has also shown a higher utilisation rate of medication in deprived neighbourhoods as compared to wealthy one in the province of Quebec [84].

### Strengths and limitations

One of the most important strengths of this study is the development of an immigration index. Although this index was developed for the metropolitan area of Montreal, the method used can easily be translated to other metropolitan areas in Canada and elsewhere. Also, the fact that we combined the immigration index with material deprivation and social deprivation indices allowed us to distinguish between “deprived immigrant neighbourhoods” and “wealthy immigrant neighbourhoods”. Another strength consists of the large number of patients living with diabetes included in the cohort (n = 111,556) and the representation of a real-world situation.

However, it is important to keep some limitations in mind. First, since immigrant status, language preferences, and socioeconomic information are not available at the individual level in administrative health data, we used immigration and socioeconomic indices at the neighbourhood-level, which may lead to some ecological bias. Also, we limited the immigration index to six neighbourhood-level variables (% of people with maternal language other than French or English, % of people who do not speak French or English, % of recent immigrants (time since immigration < 5 years), % of immigrants with time since immigration between 5 and 10 years, % of immigrants with time since immigration between 10 and 15 years, % of immigrants with time since immigration between 15 and 25 years), excluding variables such as the ethnic composition of immigrants or country of origin. This may limit the generalizability of the findings outside the metropolitan area of Montreal. Second, the case definition of diabetes used for the selection of the study cohort (one diagnosis of diabetes during a hospitalization or three diagnoses of diabetes within one year in the physician claims database) is very specific and may not include all patients living with diabetes (many patients with diabetes remain undiagnosed [40]). In fact, patients with less than three outpatient visits with a diagnosis of diabetes within a year were not included in the analysis. This may result in a selection bias. Another important limitation is that services dispensed in local health service centers (CLSC) are not available in administrative data. Since CLSCs are often one of the first places which Quebec immigrants can visit to obtain official documents, for many families they are the primary source of information about health and services [19]. Immigrants may therefore consult more often in CLSCs than the general population, resulting in an underestimation of immigrants in our study cohort.

Further studies will be needed that incorporate various characteristics of the built environment in which people live (e.g., public open space such as parks for physical activity as well as access to healthy or junk food stores). Indeed, Paquet et al. [85] documented the impact of public open space attributes on cardiometabolic risks, whereas Daniel et al. [86] found associations between fast food density and cardiovascular mortality. Moreover, results from Paquet et al. [87] revealed that the risk of developing diabetes was lower for people in areas with larger public open spaces and greater walkability.

## Conclusions

In this study, we show that patients with diabetes of the Montreal metropolitan area living in neighbourhoods with a high immigration score were less likely to die, to suffer a CVD event, to frequently visit general practitioners, but more likely to frequently visit emergency departments, specialists and to use an antidiabetic drug. These remained unchanged even after controlling for patient-level variables such as age, sex, comorbidities, as well as other neighbourhood attributes like material and social deprivation. Although we cannot firmly disentangle the individual versus the area-based effect of immigration, these results should be important for health care planning and public health assessment.

## Additional file

Additional file 1: Table S1. Comorbidities and their associated classification codes.
