# Supplementary material for: Neighbourhood immigration, health care utilization and outcomes in patients with diabetes living in the Montreal metropolitan area (Canada): a population health perspective
Source: BMC Health Serv Res. 2015 Apr 9;15:146. doi: 10.1186/s12913-015-0824-1 (PMC4422525; doi:10.1186/s12913-015-0824-1)
Supplement: Additional file 1: Table S1. — Comorbidities and their associated classification codes. [file 12913_2015_824_MOESM1_ESM.doc]

## Additional file 1

## Table S1. Comorbidities and their associated classification codes

| **Comorbidity** | **ICD-9** | **ICD-10** |
| --- | --- | --- |
| **Diabetes with complications** | 250.1-250.9 | E10-E14 not E10.9 |
| **Hypertension** | 401-405 | I10-I13, I15 |
| **Dyslipidemia** | 272 | E78, E752, E753, E755, E756, E770, E779, E881, E882, H026 |
| **Dementia** | 290, 291, 294 | F00-F06, F09, F10 |
| **Chronic pulmonary disease** | 491, 492, 493 | J41-J45 |
| **Connective tissue disease** | 710, 714, 725 | M05, M06, M08, M09, M12, M32-M36, L871 |
| **Ulcer disease** | 531-534 | K25-K28 |
| **Mild liver disease** | 571, 573 | K70, K71, K73-K77 |
| **Moderate to severe liver disease** | 070, 570, 572 | B15-B19, K72 |
| **Renal disease** | 403-404, 580-586 | I12-I13, N00, N01, N03-N05, N07, N08, N14, N17-N19, N150, N163, N290 |
| **Any tumor** | 140-195 | C00-C76 |
| **Leukemia** | 204-208 | C91-C95 |
| **Lymphoma** | 200-203 | C81-C86, C88, C90, C96 |
| **Metastatic solid tumor** | 196-199 | C77-C80, C97 |
